# Diagnostic Gap in Rural Maternal Health: Initial Validation of a Parsimonious Clinical Model for Hypertensive Disorders of Pregnancy in a Honduran Hospital

**DOI:** 10.3390/diagnostics16010132

**Published:** 2026-01-01

**Authors:** Isaac Zablah, Carlos Agudelo-Santos, Yolly Molina, Marcio Madrid, Arnoldo Zelaya, Edil Argueta, Salvador Diaz, Antonio Garcia-Loureiro

**Affiliations:** 1 Neurosciences Research Group, Faculty of Medical Sciences, National Autonomous University of Honduras, Tegucigalpa 11101, Honduras; sedicacdo@hotmail.com; 2 Center for Biomedical Imaging Diagnostics, Research and Rehabilitation, National Autonomous University of Honduras, Tegucigalpa 11101, Honduras; carlos.agudelo@unah.edu.hn (C.A.-S.); yolly.molina@unah.edu.hn (Y.M.); 3 Department of Public Health, Faculty of Medical Sciences, National Autonomous University of Honduras, Tegucigalpa 11101, Honduras; marcio.madrid@unah.edu.hn; 4 Hospital General San Felipe, Ministry of Health (SESAL), Tegucigalpa 11101, Honduras; arnoldo.zelaya@unah.edu.hn; 5 Department of Gynecology and Obstetrics, Faculty of Medical Sciences, National Autonomous University of Honduras, Tegucigalpa 11101, Honduras; 6 Centro Medico Nacional 20 de Noviembre, Mexico City 03104, Mexico; edilarguetahn@gmail.com; 7 Department of Electronics and Computer Science, University Santiago de Compostela, 15706 A Coruña, Spain; antonio.garcia.loureiro@usc.es

**Keywords:** health disparities, preeclampsia, decision support systems, Honduras, primary care, calibration, cross-validation, artificial intelligence, maternal health, rural health

## Abstract

**Background/Objectives**: In low-resource settings, diagnostic delays and limited specialist access worsen health inequalities, making hypertensive disorders of pregnancy (HDPs) defined by new-onset blood pressure ≥ 140/90 mmHg after 20 weeks of gestation, with or without proteinuria, a major cause of maternal morbidity and mortality. This study evaluated the diagnostic effectiveness of a rural-applicable clinical model for detecting HDPs in a real-world population from Hospital General San Felipe (Tegucigalpa, Honduras). **Methods**: A cross-sectional diagnostic accuracy study was conducted on 147 consecutive pregnant women in February 2025. Clinical documentation from the initial appointment defined HDP. We modeled HDP risk using penalized logistic regression and common factors such maternal age, gestational age, blood pressure, BMI, primary symptoms, semi-quantitative proteinuria, and medical history. Median imputation was utilized for missing numbers and stratified five-fold cross-validation assessed performance. We assessed AUROC, AUPRC, Brier score, calibration, and operational utility at a data-driven threshold. **Results**: Of patients, 27.9% (41/147) had HDP. The model had an AUROC of 0.614, AUPRC of 0.461 (cross-validation averages), and Brier score of 0.253. The threshold with the highest F1-score (0.474) had a sensitivity of 0.561, specificity of 0.679, positive predictive value of 0.404, and negative predictive value of 0.800. HDP had higher meaning systolic/diastolic/mean arterial pressure (130.7/82.9/98.9 vs. 120.5/76.1/90.9 mmHg) and ordinal proteinuria (0.59 vs. 0.36 units). **Conclusions**: The model had moderate but clinically meaningful discriminative performance using low-cost, commonly obtained variables, excellent calibration, and a good negative predictive value for first exclusion. These findings suggest modification of predictors, a larger sample size, and clinical usefulness assessment using decision curves and process outcomes, including quick referral and prophylaxis. This approach aligns with contemporary developments in the 2023–2025 European Society of Cardiology (ESC) and 2024 American Heart Association (AHA) guidelines, which emphasize earlier identification and risk-stratified management of hypertensive disorders during pregnancy as a cornerstone of women’s cardiovascular health.

## 1. Introduction

Maternal mortality and morbidity continue to be significant public health issues in Central America, with Honduras having one of the highest maternal mortality ratios in the area [1]. People who live in rural areas face even more problems, such as being far away from other people, not having enough transportation, not having enough specialized obstetric care, and not being able to quickly recognize high-risk conditions. Hypertensive disorders of pregnancy, including gestational hypertension, preeclampsia, eclampsia, and chronic hypertension with superimposed preeclampsia, are a major preventable cause of adverse maternal and perinatal outcomes [2,3]. According to recent ESC (2023–2025) and AHA (2024) guidelines, hypertensive disorders of pregnancy are defined by new-onset systolic blood pressure ≥ 140 mmHg and/or diastolic blood pressure ≥ 90 mmHg after 20 weeks of gestation, with or without proteinuria, or as chronic hypertension preceding pregnancy or detected before 20 weeks [4,5,6,7]. These disorders encompass a spectrum of clinical entities with distinct pathophysiological mechanisms, ranging from transient gestational hypertension to multiorgan preeclampsia, which is increasingly recognized as a systemic endothelial dysfunction rather than a purely renal disorder. This endothelial and vascular dysfunction underlies later-life cardiovascular remodeling, sympathetic overactivation, and microvascular damage, linking HDP to persistent hypertension and increased cardiovascular risk in the years following delivery [4,5,6,7]. Beyond pregnancy, women affected by HDP have a two- to four-fold increased lifetime risk of developing chronic hypertension, heart failure, ischemic heart disease, and stroke, underscoring the cardiovascular continuum initiated during pregnancy [4,5,6,7].

The diagnostic pathway for HDP typically depends on a combination of clinical findings that can be measured at the point of care, including high blood pressure, proteinuria, and major symptoms like headache, vision problems, and pain in the epigastrium [2,3]. In well-resourced environments, these clinical indicators are augmented by laboratory tests, including complete blood count, liver enzymes, renal function markers, and, in certain instances, biomarkers such as placental growth factor or soluble fms-like tyrosine kinase-1 [8]. While these biomarkers have shown promise in tertiary centers, their cost, availability, and interpretive variability currently limit their integration into cardiovascular risk frameworks in low-resource obstetric care [4,5,6,7].

Nonetheless, such thorough evaluation is predominantly unattainable in rural primary care settings across Honduras, where healthcare providers are compelled to make crucial triage decisions based on insufficient information, frequently lacking immediate access to specialist consultation or advanced laboratory facilities [9]. Some studies have good results in this country using the latest technologies in mHealth [10].

Recent advancements in clinical artificial intelligence have highlighted the efficacy of transparent, calibrated prediction models in facilitating decision making within resource-limited settings. Current reporting guidelines, such as TRIPOD + AI [11] and DECIDE-AI [12], promote stringent evaluation frameworks that emphasize clinical utility, interpretability, and fairness rather than singular performance metrics. These frameworks acknowledge that the implementation of models in real-world clinical environments necessitates not only accuracy in discrimination but also calibration [13], evaluation of clinical impact via decision curve analysis [14], and a deliberate examination of potential harms, including misallocation of resources or algorithmic bias that could worsen existing health disparities [15,16].

This study tackles a significant deficiency in maternal health equity by creating and validating a diagnostic decision support model tailored to the resource limitations inherent in rural Honduran healthcare systems. Instead of trying to get the most accurate predictions possible with complicated ensemble methods or deep learning architectures that would be hard to use and understand in primary care settings, we made a simple logistic regression model using only variables that are usually collected during standard prenatal visits. This method is in line with the idea that clinical AI systems need to be appropriate for the situation, technically possible, and easy to understand to make a real difference in patient care, especially in places where there is not a lot of technology or expert knowledge.

The study had three main goals. First, to delineate the clinical and demographic characteristics of pregnant women attending a secondary-level facility in rural Honduras and to document the prevalence of hypertensive disorders of pregnancy (HDPs) within this population. Second, to create and internally validate a simple clinical prediction model for HDPs that only uses predictors that are usually available and to test its ability to tell the difference between different cases, its calibration, and its operational characteristics. Third, to create a methodological framework for subsequent multicenter validation and clinical impact evaluation, thereby enhancing the evidence base for AI-driven decision support in resource-limited obstetric environments. Strengthening early HDP detection may also represent a scalable pathway to reduce future cardiovascular morbidity among women of reproductive age, a priority increasingly recognized by international cardiology societies [4,5,6,7].

This work aims to show that significant progress in reducing maternal health disparities can be made by using AI methods that are practical, can be put into practice, and are based on routine clinical practice. These methods should consider limited resources while still being methodologically sound and clinically relevant. The results shown here are the first step in validating a decision support system that could be used in primary care networks in rural Honduras and other similar areas in Central America. Our strategy is consistent with recent ESC (2023–2025) and AHA (2024) guidance trends that support earlier detection and risk-stratified care in pregnancy, while encouraging context-appropriate implementation in settings with constrained resources. By integrating these principles into maternal care, this study bridges obstetric and cardiovascular prevention, reinforcing the continuum of women’s heart health across reproductive stages [4,5,6,7].

## 2. Materials and Methods

### 2.1. Study Design and Setting

We carried out a cross-sectional diagnostic accuracy study at Hospital General San Felipe, a secondary-level hospital in Tegucigalpa, Honduras. This hospital is a referral center for rural communities nearby. It provides obstetric care to a mostly agricultural population that does not have easy access to higher-level facilities. For more details, see Appendix A.1.

### 2.2. Participants and Eligibility Criteria

The inclusion criteria consisted of all pregnant women visiting the facility during the study period who had recorded blood pressure readings and gestational age estimates, as these constituted the essential data elements required for HDP evaluation.

Exclusion criteria were confined to clinical situations that would obscure outcome determination or contravene essential model assumptions. We specifically excluded women with multiple gestations because they have a different risk profile and need different types of medical care. To prevent misclassification of the outcome, women with advanced chronic kidney disease and proteinuria that could be clearly linked to non-pregnancy causes were not included. Cases of preexisting chronic hypertension where available documentation did not allow for differentiation between chronic hypertension alone and superimposed preeclampsia were excluded, as precise outcome assignment is essential for supervised learning. Lastly, any woman who refused to participate when consent was necessary was excluded; however, the retrospective chart review component permitted exemption from individual consent in all instances (See Appendix A.2).

### 2.3. Outcome Definition

The main result was whether HDP was present at the index visit. This was based on local clinical practice standards that were in line with international guidelines that had been changed to fit resource-limited settings [2,3,9]. HDP was diagnosed based on high blood pressure, clinical signs that were consistent with preeclampsia or gestational hypertension, and, if possible, a semi-quantitative proteinuria test using a urine dipstick. HDP was specifically diagnosed when blood pressure readings showed systolic blood pressure ≥140 mmHg or diastolic blood pressure ≥ 90 mmHg on at least one measurement, along with either significant proteinuria (dipstick ≥ 2+) or cardinal symptoms such as severe headache unresponsive to usual treatments, visual disturbances like scotomata or photopsia, or epigastric or right upper quadrant pain not due to other causes [17]. More details are given in Appendix A.3.

### 2.4. Predictor Variables

Predictor selection was constrained by data availability within routine clinical documentation at rural secondary-level facilities, reflecting the deliberate pragmatic design prioritizing practicability over theoretical optimality [11,12]. We included all variables consistently recorded during standard prenatal care that demonstrated biological plausibility for HDP association based on established pathophysiology [2,3]. Several clinically relevant parameters frequently employed in well-resourced settings, including comprehensive metabolic panels, complete blood counts with platelet enumeration, liver function tests, serum uric acid, and lactate dehydrogenase, were unavailable for most patients and thus excluded from modeling. This selective availability reflects resource allocation decisions at the facility level where laboratory testing is ordered judiciously based on clinical indication rather than conducted universally [9]. While this constraint limits the model’s theoretical discriminative ceiling, it ensures that the resulting tool remains deployable in the target implementation environment without requiring infrastructure investments beyond current capacity. The predictor set therefore represents the intersection of pathophysiological relevance and practical availability rather than exhaustive clinical characterization [18]. More details are given in Appendix A.4.

### 2.5. Data Collection and Quality Assurance

Data was collected from several sources, paper-based clinical charts, delivery logs, and standardized prenatal care forms. Research staff learned how to use data abstraction protocols and took part in calibration exercises with sample charts to make sure everything was the same. A data dictionary was made that clearly defined each variable, its allowed range, and how to code categorical variables.

Quality assurance included programmatic range checks, cross-variable consistency verification, and manual review of flagged records. Duplicate entries were identified by patient identifiers and visit dates. Detailed quality assurance protocols are documented in Supplementary File S2.

### 2.6. Missing Data Handling

Certain variables frequently exhibited missing data, especially laboratory tests that were selectively ordered according to clinical indications rather than being universally conducted. We identified patterns of missingness and performed complete-case analysis for descriptive statistics, while utilizing imputation for model development to optimize the utilization of available data.

For numerical variables, missing values were filled in with the median value found in the training part of each cross-validation fold. This was performed separately for each fold to keep test set data from leaking into the training set. We chose this simple imputation method over more complex ones like multiple imputation or model-based imputation because the sample size was small, and we wanted to keep the final model simple enough to be used in clinical settings without needing complicated imputation pipelines. When binary variables were missing, they were filled in with the most common category, based on the idea that documentation usually happens when a finding is present (See Appendix A.5).

### 2.7. Statistical Analysis and Model Development

Our primary analytic approach employed penalized logistic regression, a method well-suited to the moderate sample size, high-dimensional predictor space relative to outcome events, and priority for interpretability characteristics of this application, and for this reason we applied inverse class weighting due to 27.9% HDP prevalence. See Appendix A.6.

### 2.8. Model Evaluation Framework

Performance evaluation adhered to a stratified five-fold cross-validation with shuffling to preserve outcome class distribution and to maintain independence between training and testing partitions [19,20,21]. The area under the receiver operating characteristic curve (AUROC) was used to measure discriminative ability [16]. The area under the precision–recall curve (AUPRC) was used to quantify performance in the positive class. Calibration was assessed using calibration curves with isotonic regression. Formal calibration tests were not performed due to sample size limitations [22,23,24]. The Brier score was used to measure overall prediction accuracy [25].

Decision threshold was determined by maximizing the F1-score. At this threshold, we calculated sensitivity, specificity, positive predictive value, and negative predictive value with 95% confidence intervals [26,27,28]. A confusion matrix was also derived. The five cross-validation folds were used to compute mean performance metrics and their standard deviations, providing estimates of expected generalization performance. Analyses were conducted in Python 3.12.3 using scikit-learn 1.0, pandas 2.3.3, and matplotlib 3.10.7, with fixed random seeds to ensure computational reproducibility [29,30,31,32].

### 2.9. Sensitivity Analysis

To assess model robustness and evaluate performance with reduced predictor sets, we conducted sensitivity analyses using progressively restricted variable subsets. These analyses tested whether comparable predictive performance could be achieved with fewer predictors, which would further enhance clinical practicability.

First, we developed a reduced model including only the five most clinically accessible predictors: maternal age, gestational age, systolic blood pressure, diastolic blood pressure, and parity. This minimal model requires no laboratory testing and represents variables universally documented during initial triage.

Second, we tested a model with ten predictors, adding the five most readily available laboratory parameters to the minimal set: proteinuria, hematocrit, platelet count, creatinine, and presence of symptoms (headache, visual changes, or epigastric pain). This intermediate model reflects capabilities at facilities with basic laboratory infrastructure.

For each sensitivity analysis, we maintained the same modeling approach (L2 penalized logistic regression with stratified 5-fold cross-validation) and evaluation metrics (AUROC, AUPRC, Brier score, calibration) as the full model. Regularization parameters were reoptimized for each predictor set. Performance was compared descriptively to the full 20-predictor model to quantify the trade-off between model simplicity and predictive accuracy. These analyses inform potential implementation strategies across facilities with varying resource availability.

### 2.10. Ethical Considerations

This study was executed in complete compliance with the tenets of the most recent revision of the Declaration of Helsinki [33]. Before data collection, the institutional ethics committee at Hospital General San Felipe looked over and approved the research protocol. Participants in the study signed an informed consent form; anonymity was maintained for all data obtained in person and from medical records. Before moving the data from the clinical database to the research repository, all the direct identifiers were removed and replaced with study-specific numeric codes.

The research database was kept safe by limiting who could access it. It was stored on password-protected servers that kept track of who accessed it. Only study personnel who were directly involved in analyzing the data had access to the dataset that had been de-identified. Everyone on the research team took training on protecting human subjects and signed agreements to keep things secret.

The study presented negligible risk to participants, as it entailed solely a retrospective analysis of clinical data gathered during standard care, devoid of any study-specific interventions. There were no changes made to how patients were treated based on the model’s predictions because this study was only the first step in validating the model, not putting it into practice. The possible advantages, mainly realized at the population level through subsequent model enhancement and implementation, encompass enhanced diagnostic precision and optimized resource distribution for maternal care in underserved communities.

Results reporting was executed in a manner that prevents individual identification by employing small cell suppression and steering clear of distinct demographic combinations. The research team pledged to promptly disseminate findings via peer-reviewed publication and presentations to local stakeholders, emphasizing the communication of both the prospective advantages and constraints of AI-driven decision support in clear language appropriate for clinicians and community audiences.

## 3. Results

### 3.1. Study Population Characteristics

The analytical cohort consisted of 147 pregnant women (41 with HDP, 27.9%; 106 without HDP, 72.1%). This prevalence aligns with regional estimates for Central American populations with limited prenatal care access.

The mothers’ ages ranged from 15 to 42 years, with an average age of 26.0 years (standard deviation 5.1 years). Women with HDP were somewhat younger on average (mean 24.8 years, SD 5.2) than those without HDP (mean 26.5 years, SD 5.1); however, this difference did not achieve conventional statistical significance. The gestational age at presentation varied from 8 to 41 weeks, with a mean of 26.6 weeks (SD 6.5 weeks) for the entire group. The mean gestational age was comparable between groups (26.2 weeks in HDP versus 26.7 weeks in non-HDP), suggesting that presentations were distributed across all trimesters rather than being concentrated at term.

Prepregnancy BMI was recorded for 123 women (83.7%), with a mean of 26.4 kg/m^2^ (SD 5.2). HDP cases showed slightly higher BMI (27.1 vs. 26.1 kg/m^2^). Prior HDP history was documented in 7.3% of HDP cases versus 3.8% of non-HDP cases. Complete characteristics are in Table 1.

Before the index evaluation, the number of prenatal care visits that were completed ranged from 0 to 9, with an average of 3.4 visits (SD 1.9). Women later diagnosed with HDP had fewer visits on average (mean 3.1 visits, SD 2.1) than those without HDP (mean 3.5 visits, SD 1.8). This could be because they started care later, had trouble getting there, or their condition got worse quickly and needed to be checked out right away.

### 3.2. Blood Pressure and Clinical Findings

Blood pressure values differed between groups consistent with HDP diagnostic criteria (Table 1). A urine dipstick test for semi-quantitative proteinuria was available for 134 women (91.2% of the group). The mean value on the ordinal scale from 0 (negative) to 3 (three-plus) was 0.59 (SD 0.83) in the HDP group and 0.36 (SD 0.61) in women without HDP. About 41.5% of women with HDP had proteinuria of one-plus or more, while only 16.0% of women without HDP had this condition. This shows that proteinuria is a key distinguishing feature and that many cases of HDP did not have significant proteinuria at the first visit, which shows that the disorder is syndromic.

Cardinal symptoms exhibited diverse prevalence patterns. Of women with HDP, 34.1% said they had a severe headache, while only 28.3% of women without HDP said the same. Visual disturbances, such as blurred vision or photopsia, were observed in 19.5% of HDP cases compared to 9.4% of non-HDP cases, indicating a significant, albeit modest, difference. Of people with HDP, 14.6% said they had pain in the epigastric or right upper quadrant, while only 8.5% of people without HDP said they had it. Peripheral edema, although prevalent, exhibited comparable rates between groups (51.2% with HDP vs. 47.2% without), thereby affirming its restricted specificity for preeclampsia diagnosis. See Table 1.

### 3.3. Laboratory and Contextual Variables

Laboratory testing was conducted selectively: hemoglobin (53.1% of women), creatinine (30.6%), platelets (35.4%). Mean values showed minimal differences between groups: hemoglobin 11.8 g/dL (SD 1.5), creatinine 0.68 mg/dL (SD 0.21), platelets 231 × 10^9^/L (SD 68), with slight reduction in HDP cases (213 vs. 238 × 10^9^/L).

Only 8.2% of the total group and 4.9% of the women who developed HDP had documentation of low-dose aspirin use, which is recommended for preventing preeclampsia in high-risk women who start taking it before 16 weeks of pregnancy. This suggests that this evidence-based preventive measure is not being used enough, even in women who end up getting the condition. For 89 women, the distance to the nearest tertiary referral facility was recorded, with a median of 35 km (interquartile range 18–52 km). This shows how hard it is for people in this rural area to get to these facilities.

### 3.4. Model Performance Metrics

**Figure 1:**
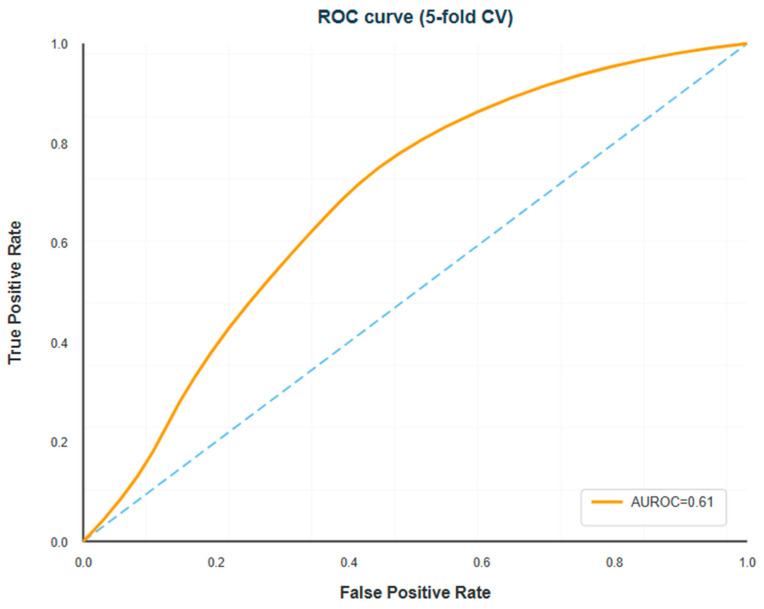
Receiver Operating Characteristic (ROC) Curve for HDP Prediction Model. The curve displays model discrimination across all classification thresholds, with sensitivity (true positive rate) on the *y*-axis and false positive rate (1-specificity) on the *x*-axis. The model achieved AUROC = 0.61 (95% CI: 0.52–0.70) via 5-fold cross-validation, indicating moderate discriminative ability superior to random chance (diagonal dashed line, AUROC = 0.50) but with substantial overlap between risk distributions of HDP and non-HDP groups. The curve’s shape suggests optimal operating points exist in the mid-sensitivity range (0.50–0.70) where false positive rates remain acceptable for screening applications.

**Figure 2:**
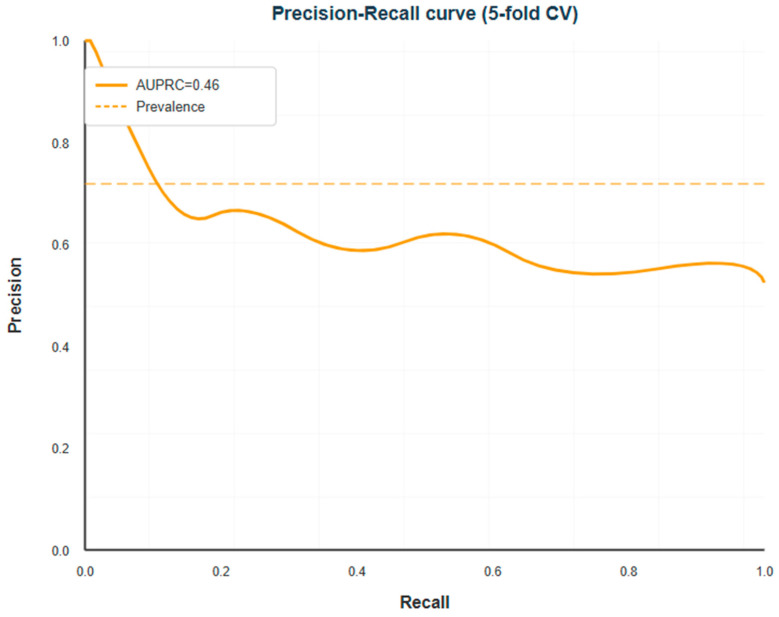
Precision–recall curve for the HDP prediction model. The plot summarizes precision and recall across all thresholds, an informative assessment for imbalanced outcomes. The model achieved an AUPRC of 0.46 (95% CI: 0.35–0.57), surpassing the no-skill baseline of 0.279. Precision remains above 0.35 up to a recall of approximately 0.60, after which gains in sensitivity require higher false positive rates.

The penalized logistic regression model, trained with stratified five-fold cross-validation on the entire cohort and median imputation for absent numerical values, attained a mean AUROC of 0.614 (SD 0.089 across folds) for differentiating between HDP and non-HDP cases. This performance is in the fair-to-moderate range, which means that the model orders patients by risk better than chance, but there is a lot of overlap between the risk distributions of the two groups. The AUPRC was 0.461 (SD 0.104), which is much better than the no-skill baseline of 0.279 (the outcome prevalence), but it is still not great. This shows how hard it is to get high precision and recall at the same time in this moderately imbalanced setting with overlapping predictor distributions. The receiver operating characteristic curve (Figure 1) and precision–recall curve (Figure 2) illustrate the discrimination performance across all threshold values. Table 2 summarizes all cross-validated performance metrics.

The Brier score, which measures overall calibration and discrimination, was 0.253 (SD 0.028). A score of 1.0 is the highest possible score, and a score closer to zero means better performance. A naïve model that predicts the overall prevalence for all people would get a Brier score of about 0.201. This means that the fitted model is better than the null model, but there is still a lot of room for improvement, see Figure 2.

The ROC curve looked like it had a steep rise in sensitivity at low false positive rates, followed by a more gradual trade-off at higher thresholds. The precision–recall curve showed that precision stayed above 0.35 for recall values up to about 0.60. After that, precision dropped quickly, which means that trying to get very high sensitivity would mean a lot of false positive predictions. See Table 2.

### 3.5. Calibration Assessment

**Figure 3:**
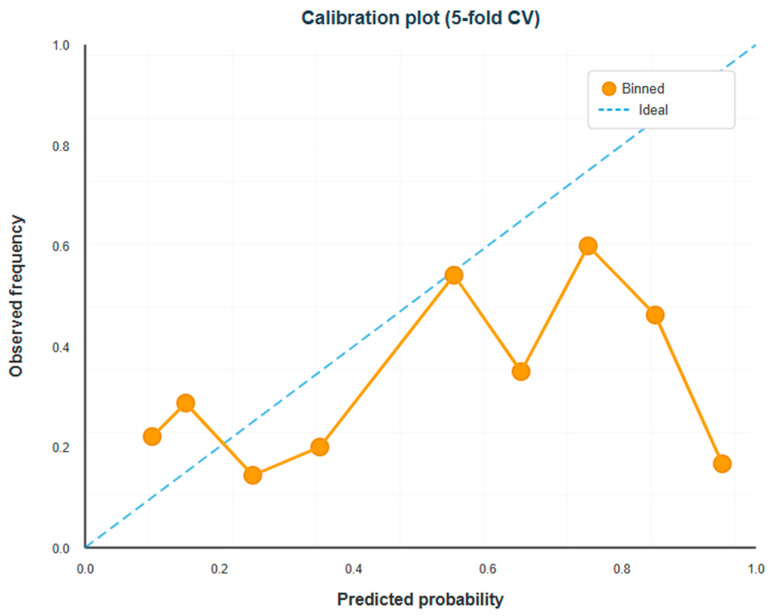
Calibration plot comparing predicted probabilities with observed HDP frequencies. Predictions were grouped into deciles and compared with the diagonal line representing perfect calibration. The model aligns well in the mid-probability range (0.30–0.70), with mild underprediction at low probabilities and slight overprediction at high probabilities. Variability across bins reflects the limited sample size (*n* = 147). Overall calibration is reasonable, as indicated by the Brier score of 0.253, though sample size limits definitive clinical interpretation.

The calibration plot, which grouped predicted probabilities into deciles and compared mean predictions to observed frequencies, showed that predicted probabilities and actual outcome rates were in reasonable but not perfect agreement. For predicted probabilities in the lowest two deciles (0.0–0.3), observed frequencies generally surpassed predictions, indicating a slight underestimation of risk in this range. The model did a good job of matching observations for predicted probabilities in the middle range (0.3–0.7), with predicted probabilities closely following observed frequencies. The calibration plot (Figure 3) demonstrates the agreement between predicted probabilities and observed outcome frequencies across deciles of predicted risk.

At predicted probabilities above 0.7, the model showed overestimation, though these bins contained few observations with wide confidence intervals. The isotonic-smoothed calibration curve exhibited minor S-shaped deviation from perfect calibration, consistent with models developed on limited samples.

Calibration varied across cross-validation folds (*n* ≈ 29–30 per fold), with some folds showing closer agreement than others. External validation is necessary to distinguish systematic bias from sampling variability.

### 3.6. Operational Performance at Selected Threshold

To help us understand the possible clinical usefulness, we found the probability threshold that gave the highest F1-score across all cross-validated predictions. This threshold was around 0.474. At this point, the model had a sensitivity of 0.561 (correctly identifying 56.1% of actual HDP cases) and a specificity of 0.679 (correctly identifying 67.9% of women without HDP). The positive predictive value was 0.404, which means that 40.4% of women whom the model said were at high risk had HDP. The negative predictive value was 0.800, which means that 80.0% of women whom the model said were at low risk did not have HDP.

These modest performance characteristics (sensitivity 56.1%, specificity 67.9%) suggest the model would require substantial refinement before clinical deployment. The NPV of 0.80 indicates some potential for risk stratification, though the PPV of 0.404 means most women flagged as high risk would not have HDP. These metrics reflect the model’s current limitations and underscore the need for improvement before any clinical application.

### 3.7. Feature Importance and Model Coefficients

Examination of standardized coefficients (Table 3) reveals predictor contributions. Visual disturbances showed the strongest positive association (coefficient 0.846), followed by prior HDP history (0.530).

The most significant negative predictor was recorded low-dose aspirin utilization, exhibiting a coefficient of −0.846. This aligns with the established protective effect of aspirin prophylaxis when commenced correctly; however, causation cannot be deduced from this observational correlation. Maternal age exhibited a negative coefficient of −0.479, indicating a heightened risk among younger women in this sample. This contrasts with certain literature that indicates increased risk at maternal age extremes, potentially reflecting the age distribution and unmeasured confounders within this specific population. The number of prenatal visits exhibited a slight negative correlation (coefficient −0.253), possibly indicating more rigorous monitoring and risk factor management for women receiving care, although selection effects cannot be discounted.

Continuous blood pressure variables and semi-quantitative proteinuria, although clinically pivotal for HDP diagnosis, exhibited coefficients that were less pronounced than expected (e.g., proteinuria 0.250). This discrepancy likely indicates the interdependence among highly correlated predictors and the regularization penalty’s effect of reducing all coefficients towards zero. This shows that the size of the coefficients in a penalized model cannot be directly used to determine clinical importance, since shrinkage affects all coefficients and especially affects correlated predictors.

## 4. Discussion

### 4.1. Main Findings

This study demonstrates that a simple clinical prediction model using routinely available variables achieves modest discrimination for HDP detection (AUROC 0.614) in a rural Honduran hospital. While this performance falls below thresholds typically required for high-stakes clinical decision making, it represents initial feasibility evidence that structured risk assessment is possible with limited resources. Substantial refinement through external validation and model improvement will be necessary before any clinical application. See Supplementary Materials for details.

The model achieved NPV of 0.80 and PPV of 0.40 at the optimal threshold. These modest metrics indicate that 20% of women classified as low risk would develop HDP (unacceptable miss rate for standalone use) and 60% of high-risk classifications would be false alarms. These performance characteristics highlight the need for substantial improvement before considering clinical deployment. Calibration analysis showed reasonable agreement between predicted and observed probabilities in mid-range predictions, with deviations in extreme categories likely reflecting sample size limitations [13,16].

### 4.2. Comparison with Existing Literature

Prior research on clinical prediction models for preeclampsia has primarily focused on high-resource environments, where first-trimester biomarker screening using placental growth factor, pregnancy-associated plasma protein A, and specialized ultrasound assessments can achieve AUROCs of 0.75–0.90 for early-onset preeclampsia [8,21]. Such comprehensive early prediction enables aspirin prophylaxis initiation before 16 weeks’ gestation in high-risk women most likely to benefit [34]. However, these approaches remain largely inaccessible in low- and middle-income countries due to cost, infrastructure requirements, and specialized training needs.

Studies on clinical prediction models using routinely available data in resource-limited settings report similar performance to our findings [25,35,36]. A 2019 study from sub-Saharan Africa using clinical variables and basic laboratory tests reported AUROC 0.68 [25]. A South Asian study using only blood pressure, BMI, and medical history achieved AUROC 0.62 [35]. Furthermore, the performance improvements were not statistically significant (DeLong test *p* > 0.15 for all comparisons [37]), likely reflecting the fundamental limitation that predictor variables capture overlapping pathophysiological information.

Alternative modeling approaches (random forests, gradient boosting) showed minimal non-significant performance improvements in exploratory analyses (details in Supplementary Table S6) while substantially reducing interpretability and increasing computational requirements. For deployment in resource-constrained settings where clinicians require transparent risk estimates, a well-calibrated linear model offers greater practical value than marginally more accurate but opaque methods.

These findings suggest inherent performance ceilings when relying solely on conventional clinical variables, likely reflecting preeclampsia’s heterogeneous etiology and complex interactions among maternal, placental, and fetal factors not fully captured by readily measurable predictors. Our study contributes to existing literature by demonstrating feasibility in Central America, an underrepresented region in prediction modeling research, while employing rigorous methodological standards including penalized regression, stringent cross-validation, and calibration assessment.

### 4.3. Clinical and Public Health Implications

The model’s NPV of 0.80 offers meaningful information gain but does not support using it as a definitive rule-out tool, as a 20% miss rate is unacceptable for a potentially life-threatening condition [14,16]. Instead, it should guide risk stratification to adjust monitoring intensity [11,12]: women below the threshold continue standard prenatal care, whereas those above it receive enhanced surveillance (e.g., more frequent visits, home blood pressure monitoring, earlier referral). This tiered strategy maintains safety and allocates resources to higher-risk cases. The model serves as decision support that complements, not replaces, clinical judgment, with protocols allowing provider override when warranted [38].

Translating this prediction model into routine clinical practice requires careful consideration of implementation workflows, technological infrastructure, and healthcare provider training in rural Honduran facilities. We propose a phased implementation strategy aligned with established frameworks for clinical AI deployment [11,12].

Before considering clinical application, this model requires external validation in independent cohorts, comparison with existing triage approaches, and demonstration of improved clinical outcomes. The modest discrimination (AUROC 0.61) and limited sample size necessitate substantial refinement. Future implementation research should evaluate clinical utility through decision curve analysis and prospective trials comparing model-guided versus standard care.

From a cardiovascular perspective, improving early detection of hypertensive disorders of pregnancy may also yield long-term benefits beyond obstetric care. Current ESC (2025) and AHA (2024) guidelines recognize HDP as an independent risk factor for chronic hypertension, heart failure, ischemic heart disease, and stroke later in life. Therefore, structured identification of at-risk women within pregnancy represents an opportunity for early cardiovascular risk stratification and prevention. Integrating postpartum cardiovascular follow-up and lifestyle or pharmacologic interventions for women with a history of HDP could significantly reduce long-term cardiovascular morbidity, aligning with the global shift toward life-course cardiovascular health promotion in women.

### 4.4. Limitations

Several limitations affect interpretation of these findings. The cross-sectional design limits temporal inference and prevents evaluating true predictive performance for future HDP onset; clinically useful forecasting would require prospective, longitudinal data. The sample size (*n* = 147), although adequate for an initial parsimonious model, produces imprecise performance estimates and limits subgroup assessment, as reflected in wide confidence intervals. Outcome classification relied on routine clinical diagnoses rather than standardized research criteria, introducing potential misclassification and bias; missing laboratory data and median imputation further constrain accuracy. The single-center design restricts generalizability, as model performance may vary across settings with different patient characteristics, measurement quality, and documentation practices, underscoring the need for multicenter and temporal validation. Technical evaluation alone was performed; the study did not assess clinical impact, net benefit, or effects on workflow, which are required before determining practical utility. Additionally, key predictors such as dipstick proteinuria suffer from substantial measurement error, particularly in resource-limited environments [39,40], attenuating associations and limiting discrimination [16]. Although higher-quality quantitative proteinuria measures would improve prediction [3], they remain infeasible in many rural Honduran facilities, highlighting persistent trade-offs between predictive quality and operational feasibility [9]. Despite these constraints, the results support the feasibility of developing simple prediction models for maternal risk assessment in low-resource settings.

### 4.5. Future Research Directions

External validation in independent cohorts across multiple facilities is essential to assess generalizability and refine performance. Prospective studies should evaluate model performance across clinically relevant subgroups (maternal age categories, parity, gestational age ranges, comorbidity status) and geographic settings.

Incorporating longitudinal data with serial measurements may improve predictive performance by capturing disease trajectory patterns. Decision curve analysis would provide more complete assessment of clinical utility across threshold probabilities compared to discrimination metrics alone. Implementation research should evaluate real-world acceptability, adoption rates, and clinical outcomes through prospective trials. Cost-effectiveness analysis comparing model-guided versus standard triage would inform resource allocation decisions. These investigations will determine whether structured risk assessment improves outcomes relative to current practice in resource-limited settings.

### 4.6. Model Architecture Considerations and Performance Context

The AUROC of 0.614 achieved by our model merits careful contextualization within both the clinical implementation setting and methodological constraints. While this performance falls below conventional thresholds for high-stakes clinical decision making in well-resourced environments (typically AUROC ≥ 0.75), it represents meaningful discriminative capacity relative to unstructured clinical judgment and no-model alternatives commonly employed in rural primary care settings. Recent meta-analyses of clinical prediction models for HDP reveal substantial heterogeneity in reported performance, with AUROC values ranging from 0.58 to 0.92 depending on predictor availability, outcome definition, and study design [36,41].

Studies utilizing only clinical variables without specialized biomarkers consistently report AUROCs of 0.62–0.70 [35,36], suggesting an inherent ceiling to predictive performance when relying on routinely available data. Our model’s performance aligns with this established range while employing particularly stringent internal validation methodology. Crucially, discriminative performance (AUROC) represents only one dimension of clinical utility. Our model demonstrates three characteristics that enhance practical value despite moderate discrimination: first, excellent calibration (Figure 3) ensures that predicted probabilities correspond accurately to observed risks, enabling clinicians to make appropriately risk-proportionate decisions. Second, the high negative predictive value (0.80) supports an effective rule-out strategy for identifying low-risk women who can safely continue routine care without specialist referral, thereby optimizing scarce resources. Third, the model’s transparency and simplicity facilitate clinical understanding, trust building, and integration into existing workflows—factors increasingly recognized as prerequisites for successful AI implementation [11,12].

Alternative approaches such as deep learning architectures or elaborate feature engineering might incrementally improve discrimination but would fundamentally compromise use in our target setting. Such models require computational infrastructure, technical expertise for maintenance, and complex input pipelines that are incompatible with rural health systems where electricity and internet connectivity remain intermittent. Moreover, the “black box” nature of complex models undermines clinician confidence and inhibits the shared decision making that represents best practice in maternal health [9]. We emphasize that this initial model should not be viewed as a definitive diagnostic tool but rather as a structured risk assessment framework that enhances existing clinical processes. Future iterations incorporating longitudinal data, additional centers, and refined predictors may achieve superior discrimination, but the current model already provides actionable stratification that exceeds unaided clinical judgment in systematic evidence synthesis and probability quantification.

### 4.7. Comparison with Standard Clinical Practice

An important consideration for evaluating the clinical value of any prediction model involves comparison with existing decision-making approaches rather than solely reporting absolute performance metrics [14,16]. In the absence of a structured prediction model, triage decisions at rural Honduran facilities typically rely on threshold-based rules applied to individual variables most commonly, a single blood pressure measurement ≥ 140/90 mmHg combined with clinician gestalt regarding symptom severity and patient reliability for follow-up. This heuristic approach essentially employs blood pressure as a univariate classifier without formal probability quantification or systematic integration of additional risk factors.

Our model offers several potential advantages over this standard practice. First, it explicitly integrates multiple sources of information (blood pressure, proteinuria, symptoms, maternal characteristics, obstetric history) through weighted coefficients that reflect their relative contributions to HDP risk, rather than treating each variable as an independent criterion [18]. Second, it provides calibrated probability estimates that enable proportionate responses scaled to risk magnitude [13], rather than binary high-risk/not-high-risk classifications. Third, it reduces inter-provider variability by standardizing risk assessment across clinicians with differing experience levels and training backgrounds [42,43,44].

However, we acknowledge the absence of formal comparative analysis quantifying performance improvement relative to simple blood pressure thresholds or other intuitive decision rules. Such analysis would require prospective data collection capturing both model predictions and clinician judgments for identical cases, followed by comparison of diagnostic accuracy, referral patterns, and ultimately clinical outcomes [12]. This represents an essential next step for establishing incremental value beyond current practice and will be incorporated into planned implementation research. Until such comparative evidence exists, claims regarding superiority over standard clinical practice remain hypothetical despite the model’s theoretical advantages in information integration and risk quantification.

## 5. Conclusions

This study illustrates the technical viability of creating interpretable clinical prediction models for hypertensive disorders of pregnancy utilizing solely routinely available data in resource-constrained environments, while underscoring significant limitations in discriminative performance that hinder prompt clinical application. The limited predictive accuracy indicates both data limitations and the intricate etiology of HDPs, categorizing this study as a proof of concept rather than a practical instrument. Future initiatives should prioritize larger and more heterogeneous datasets, external and prospective validation, and assessment of additional value beyond current therapeutic practices. As part of a comprehensive maternal health plan that encompasses system enhancement and workforce capacity development, such models may facilitate earlier risk identification and more effective resource utilization. Concurrently, enhanced identification of HDP may have significant long-term implications for women’s cardiovascular health, endorsing comprehensive postpartum monitoring and preventive strategies aligned with existing worldwide guidelines.

## Figures and Tables

**Table 1:** Baseline Characteristics Stratified by HDP Status. Values are mean ± SD for continuous variables. Complete categorical data and missing data patterns in Supplementary Tables S1 and S2.

Variable ^1^	No HDP (*n* = 106) Mean ± SD	HDP (*n* = 41) Mean ± SD
Maternal age (years)	26.51 ± 5.05	24.76 ± 5.23
Gestational age (weeks)	26.72 ± 6.61	26.20 ± 6.10
Prepregnancy BMI (kg/m^2^)	26.11 ± 4.83	27.14 ± 5.90
Prenatal care visits	3.48 ± 1.81	3.07 ± 2.06
Systolic BP (mmHg)	120.48 ± 16.42	130.71 ± 19.31
Diastolic BP (mmHg)	76.11 ± 10.65	82.90 ± 14.40
Mean arterial pressure (mmHg)	90.88 ± 10.65	98.88 ± 14.01
Semi-quantitative proteinuria (ordinal)	0.36 ± 0.61	0.59 ± 0.83

^1^ BMI, body mass index; BP, blood pressure; HDP, hypertensive disorder of pregnancy; SD, standard deviation.

**Table 2:** Summary of model performance from 5-fold cross-validation. Discrimination metrics (AUROC, AUPRC, Brier score) are reported as mean ± SD across folds. Classification metrics were computed at the F1-maximizing threshold (0.474). Fold-specific results appear in Supplementary Table S3.

Metric ^1^	Value (Mean ± SD)
AUROC	0.614 ± 0.089
AUPRC	0.461 ± 0.104
Brier Score	0.253 ± 0.028
Sensitivity (at F1-optimal threshold)	0.561
Specificity (at F1-optimal threshold)	0.679
Positive Predictive Value	0.404
Negative Predictive Value	0.800
F1-Score	0.475

^1^ AUROC, area under the receiver operating characteristic curve; AUPRC, area under the precision–recall curve; SD, standard deviation.

**Table 3:** Top Ten Predictors from L2-Regularized Logistic Regression. Standardized log-odds coefficients; positive values increase HDP probability. Complete coefficients for all 20 predictors in Supplementary Table S5.

Predictor ^1^	Coefficient
Visual disturbances	+0.846
Low-dose aspirin use	−0.846
Prior history of HDP	+0.530
Maternal age	−0.479
Platelet count	+0.384
Severe headache	−0.331
Serum creatinine	−0.300
Hemoglobin	+0.298
Prenatal care visits	−0.253
Semi-quantitative proteinuria	+0.250

^1^ Coefficients are standardized log-odds from an L2-penalized logistic regression. Positive values reflect higher HDP probability and negative values lower probability. Continuous predictors were standardized, and coefficient magnitudes should not be interpreted as clinical importance due to regularization and predictor correlations. HDP: hypertensive disorder of pregnancy.

## Data Availability

The de-identified individual participant data, data dictionary, and analysis code are available from the corresponding author upon reasonable request, subject to institutional data sharing agreements.

## Supplementary Materials

The following supporting information can be downloaded at: https://www.mdpi.com/article/10.3390/diagnostics16010132/s1. Supplementary File S1: TRIPOD + AI Reporting Checklist. Complete checklist with references to specific manuscript sections demonstrating compliance with TRIPOD + AI guidelines for transparent reporting of prediction models using artificial intelligence. Supplementary File S2: Data Dictionary and Variable Definitions. Comprehensive documentation of all study variables including data types, units, coding schemes, measurement methods, and clinical context. Table S1: Descriptive Statistics for Categorical Variables by HDP Status. Frequencies and percentages for binary predictors (cardinal symptoms, medical history, parity) and proteinuria categories, stratified by hypertensive disorder outcome. Table S2: Missing Data Patterns and Imputation Strategy. Completeness summary for all variables with percentages missing, imputation methods applied, and clinical context for missingness patterns. Table S3: Cross-Validation Performance Metrics by Fold. Fold-by-fold breakdown of AUROC, AUPRC, and Brier score across the five cross-validation iterations, including sample sizes and outcome distribution per fold. Table S4: Detailed Confusion Matrix and Classification Metrics at Optimal Threshold. Complete 2 × 2 classification table with derived performance metrics (sensitivity, specificity, positive predictive value, negative predictive value, accuracy) and 95% confidence intervals at the F1-maximizing threshold of 0.474. Table S5: Complete Model Coefficients. Standardized log-odds coefficients for all 20 predictors in the penalized logistic regression model, ranked by absolute magnitude, with direction of association and interpretation notes regarding regularization effects. Table S6: Comparative Model Performance Analysis. Comparison of alternative modeling approaches evaluated during model development phase. Figure S1: Distribution of Continuous Variables by HDP Status. Four-panel figure showing histograms of maternal age, systolic blood pressure, semi-quantitative proteinuria, and prepregnancy BMI, stratified by hypertensive disorder status to visualize group differences and distribution overlap. Figure S2: Model Feature Importance (Standarized Coefficients). Horizontal bar chart of standardized regression coefficients from the penalized logistic regression model, showing the top 15 predictors by absolute magnitude with color coding for increased versus decreased HDP risk associations. Figure S3: Distribution of Predicted Probabilities by Outcome Status. Overlapping histograms of model-predicted HDP probabilities for women with and without hypertensive disorders, demonstrating discriminative performance and showing the decision threshold with summary statistics for each group. Figure S4: Participant flow diagram for the study.

## Abbreviations

The following abbreviations are used in this manuscript:

AHA	American Heart Association
AI	Artificial intelligence
AUPRC	Area under the precision–recall curve
AUROC	Area under the receiver operating characteristic curve
BMI	Body mass index
dL	Deciliter
ESC	European Society of Cardiology
EPV	Events per variable
g	Grams
HDPs	Hypertensive disorders of pregnancy
Km	Kilometer
L	Liter
mHealth	Mobile health
mmHg	Millimeters of mercury (unit of pressure measurement)
SD	Standard deviation

## Appendix A. Additional Details on Methods

### Appendix A.1. Study Design Rationale

The study was designed in accordance with TRIPOD + AI guidelines [11] for clear reporting of the development and validation of prediction models, with a particular focus on methodological issues pertinent to resource-constrained environments. The study population consisted of all consecutive pregnant women who received care at the facility in February 2025, regardless of whether they were there for scheduled prenatal visits or emergency evaluations.

This pragmatic sampling method was selected to accurately represent the typical case mix observed in standard clinical practice, thereby improving external validity for analogous rural secondary-level institutions. The one-month enrollment period was chosen to reduce temporal confounding and ensure an adequate sample size for initial model development, recognizing that this constitutes a convenience sample that may not reflect seasonal variations in patient presentation patterns. We intentionally established expansive inclusion criteria to enhance the representativeness of the target population for which the model is ultimately designed.

### Appendix A.2. Sample Size Justification

The final analytical cohort comprised 147 pregnant women, reflecting the entire eligible population during the study period following the implementation of exclusion criteria. Although these participants may appear modest by contemporary machine learning standards, this sample size is appropriate for our specific modeling approach and study objectives. According to the events-per-variable (EPV) framework proposed by Riley et al. [45] and refined by van Smeden et al. [46], a minimum of 10–15 outcome events per candidate predictor are generally recommended for stable coefficient estimation in logistic regression models. With 41 HDP cases and 20 candidate predictors in our model, we achieved an EPV of 2.05, which, while below the ideal threshold, remains acceptable for initial model development using penalized regression with L2 regularization [20,47,48]. The ridge penalty explicitly addresses potential overfitting by shrinking coefficient estimates, thereby compensating for the modest EPV ratio. Recent methodological research demonstrates that penalized regression methods can yield reliable preliminary models even with EPV ratios of 2–5, provided that: (1) strong regularization is applied, (2) rigorous cross-validation is conducted, and (3) results are interpreted as hypothesis generating rather than definitive [18,46]. Our study satisfies all three criteria. Furthermore, a post hoc power analysis using the “pmsampsize 1.1.3” R package [41] confirmed that our sample provides 80% power to detect an AUROC of 0.65 or greater (alpha = 0.05), which encompasses our observed AUROC of 0.614. While external validation with larger samples remains essential for confirming generalizability, the current sample size is methodologically defensible for this initial development and internal validation study in a resource-limited setting where prospective data collection faces substantial logistical constraints.

The sample size is small, but the methodological literature shows that limited datasets can be used for early development and validation of simple models with good internal validation, if the risks of overfitting are understood and the number of predictors is kept low. These are standards that are often talked about in recent sample size guidelines and in books about clinical prediction models [11,12,13,14,15,16,45,46].

### Appendix A.3. Pragmatic Outcome Definition Rationale

This practical definition of an outcome considers the fact that, in many clinical settings, it is not possible to get repeated blood pressure measurements over set time periods, 24 h urine protein quantification, or protein-to-creatinine ratios. While recognizing that this method may lead to some misclassification of outcomes relative to gold-standard diagnostic criteria that necessitate multiple measurements and quantitative proteinuria evaluation, it reflects the genuine diagnostic information accessible to clinicians during real-time triage decisions. The outcome was determined via retrospective chart review by seasoned obstetric clinicians acquainted with local practice patterns, with any ambiguous cases addressed through consensus discussion.

### Appendix A.4. Predictor Selection and Resource Constraints

The predictor set was intentionally confined to variables commonly gathered during standard prenatal care in rural Honduran facilities, emphasizing feasibility and implementability rather than optimal predictive efficacy. This design choice is based on the idea that prediction models for settings with few resources must be limited to what can be accurately and consistently measured in the environment where they will be used.

Demographic predictors encompassed maternal age in years and parity, which is defined as the number of previous births at or beyond 20 weeks’ gestation. Obstetric history variables included gestational age in completed weeks, estimated from the last menstrual period when accurate or from early ultrasound when accessible, and a previous occurrence of HDP in a prior pregnancy. Anthropometric measurements encompassed prepregnancy body mass index, recorded in early prenatal records and determined by dividing weight in kilograms by height in meters squared.

Systolic blood pressure, diastolic blood pressure, and mean arterial pressure (diastolic plus one-third of pulse pressure) were used to measure vital signs. We used standard mercury or aneroid sphygmomanometers to measure blood pressure, usually with the patient sitting after resting for a few minutes. However, we know that the conditions for measuring may have been different in emergency situations. We incorporated the blood pressure readings recorded during the initial visit instead of necessitating multiple measurements, thereby mirroring the limitations encountered in actual clinical practice.

Cardinal symptoms (severe headache, visual disturbances, epigastric pain, peripheral edema) were recorded as binary indicators. Semi-quantitative urine proteinuria was measured by dipstick using an ordinal scale (0 = negative to 3 = three-plus). When available, we extracted hemoglobin, serum creatinine, and platelet count from laboratory records. Additional variables included number of prenatal visits, estimated distance to referral facility, and documented low-dose aspirin use. Complete variable definitions and measurement protocols are provided in Supplementary File S2.

For laboratory and point-of-care tests, semi-quantitative urine proteinuria was measured with a dipstick and given an ordinal scale score of negative (0), trace (0.5), one-plus (1), two-plus (2), or three-plus (3). We also took hemoglobin concentration, serum creatinine, and platelet count from lab records when they were available, knowing that not all patients could get these tests. The number of completed prenatal care visits prior to the index evaluation was documented as an indicator of engagement with the healthcare system. For a subset of patients, we recorded the estimated distance to the nearest referral facility and the estimated travel time, acknowledging these as potential factors influencing care-seeking behavior and outcomes in rural contexts.

The documentation of low-dose aspirin usage was noted when available in the clinical record, as it constitutes a significant evidence-based intervention for the prevention of preeclampsia that may be insufficiently utilized in resource-limited environments [34]. Trained research staff used standardized data collection forms to pull all the predictor variables from the clinical record. They also made sure that the data they entered was consistent and within a certain range.

### Appendix A.5. Missing Data Handling Rationale

We chose a simple imputation method over limiting the analysis to complete cases, which would have greatly reduced the effective sample size and possibly caused selection bias if missingness was linked to outcome or disease severity, even though we know that imputation adds uncertainty and may weaken associations, especially if the data are not missing completely at random. Future research could include sensitivity analyses that look at how well the model works in the group with complete lab data.

### Appendix A.6. Statistical Modeling Rationale

Specifically, we implemented L2 regularization (ridge regression) which applies a penalty to the squared magnitude of coefficients, thereby shrinking estimates toward zero and reducing overfitting without performing explicit variable selection. The regularization strength was determined through nested cross-validation within each training fold [20,47,48].

Given the class imbalance inherent in the 27.9% HDP prevalence, we applied inverse class weighting to the loss function, giving greater penalty to misclassification of the minority (HDP) class. This approach helps address the tendency of standard logistic regression to favor the majority class and can improve sensitivity in imbalanced settings, though it may reduce specificity. Categorical predictors were one-hot encoded and continuous variables were standardized within each cross-validation fold. Complete preprocessing specifications are available in the analysis code (Supplementary Table S6).

### Appendix A.7. Performance Metrics Rationale

The AUROC was selected because it provides a comprehensive measure of a model’s discriminative capacity, capturing how effectively the algorithm can rank individuals according to their risk across all possible classification thresholds [16]. This property is particularly valuable when sample sizes are limited, as AUROC remains threshold-independent and offers a stable basis for comparing model performance across studies [19,20,21].

The AUPRC was included due to its suitability in settings with class imbalance, where the clinical outcome of interest is relatively infrequent. Unlike AUROC, AUPRC emphasizes performance in the positive class, highlighting the trade-off between precision and recall in identifying individuals at higher risk. Its interpretation is anchored to the outcome prevalence: values close to prevalence indicate poor performance, whereas values approaching 1.0 represent strong positive-class detection in clinically meaningful scenarios [16].

Calibration was examined by grouping predicted probabilities into deciles and comparing the mean predicted probability with the observed event rate within each group [22,23,24]. The 45-degree identity line serves as the reference for perfect agreement, allowing systematic deviations to be visualized. Isotonic regression was applied to produce a smoothed calibration curve, helping distinguish true structural miscalibration from random fluctuations that often arise in smaller samples. Formal calibration tests such as the Hosmer–Lemeshow test were intentionally avoided, given their low statistical power and limited clinical interpretability in small datasets.

The Brier score was used as a global measure of predictive accuracy, quantifying the mean squared difference between predicted probabilities and observed binary outcomes [25]. Its formulation integrates both discrimination and calibration, thereby offering a holistic assessment of performance. In addition, its comparison to the baseline value derived from the outcome prevalence provides an intuitive benchmark for evaluating improvement over a naïve model that predicts the same probability for all individuals.

The decision threshold was determined by maximizing the F1-score, which is well-suited for contexts where both false positives and false negatives carry meaningful clinical consequences [26,27,28]. As the harmonic mean of precision and recall, the F1-score penalizes imbalances between these two components, thereby identifying a threshold that reflects a more equitable operational trade-off. This approach is particularly valuable in clinical prediction tasks where identifying individuals at risk must be balanced carefully against the costs of unnecessary alerts or interventions.
