# Supplementary material for: Diagnostic Gap in Rural Maternal Health: Initial Validation of a Parsimonious Clinical Model for Hypertensive Disorders of Pregnancy in a Honduran Hospital
Source: Diagnostics (Basel). 2026 Jan 1;16(1):132. doi: 10.3390/diagnostics16010132 (PMC12785390; doi:10.3390/diagnostics16010132)
Supplement: Supplementary file 1 [file diagnostics-16-00132-s001.zip › figure_S1_distributions.pdf]

Supplementary Figure S1. Distribution of Continuous Variables by HDP Status

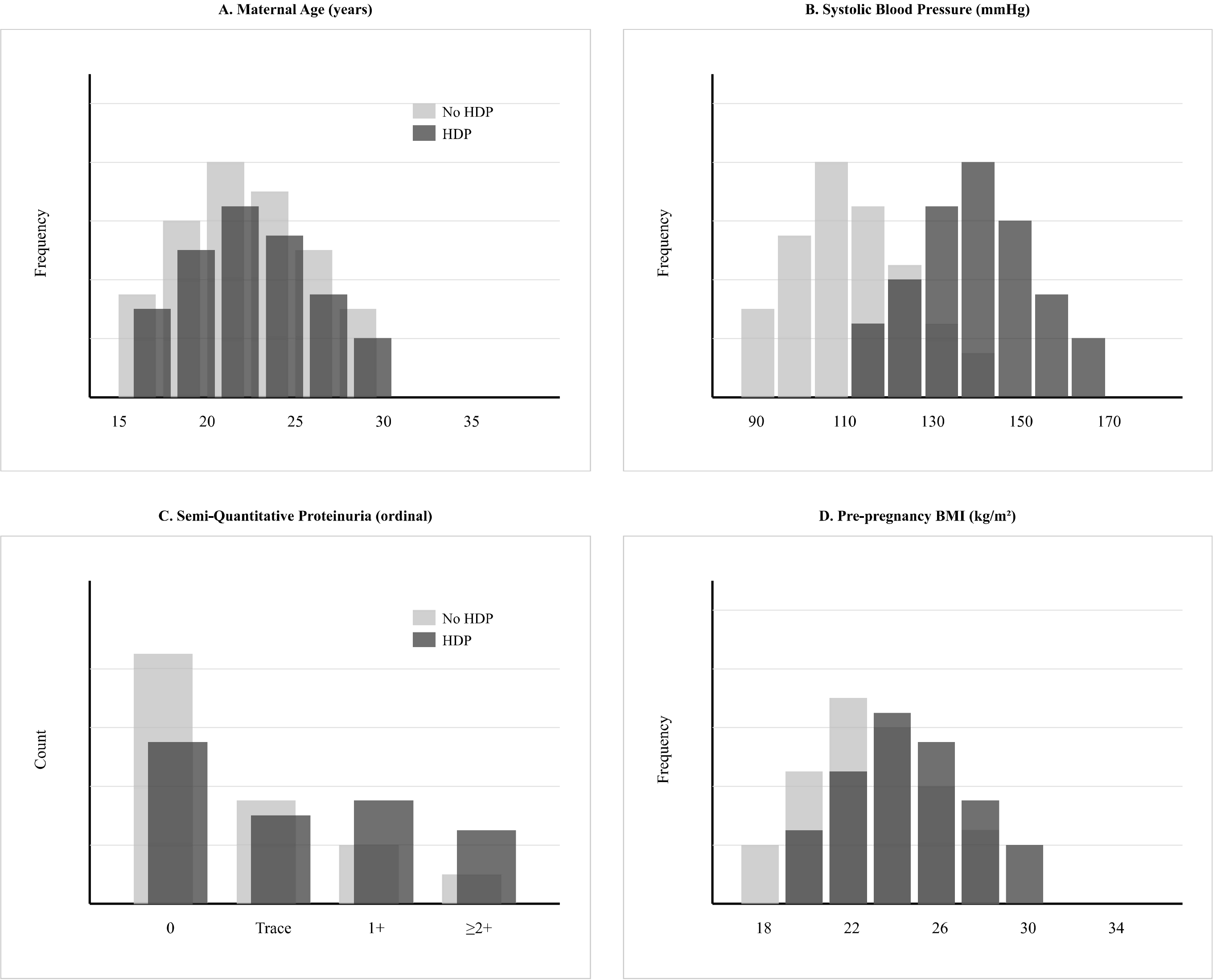

Supplementary Figure S1. Distribution of continuous variables stratified by hypertensive disorders of pregnancy (HDP) status. **(A)** Maternal age shows slightly lower mean in HDP group (24.8 vs 26.5 years). **(B)** Systolic blood pressure demonstrates clear separation between groups, with HDP showing higher mean (130.7 vs 120.5 mmHg). **(C)** Semi-quantitative proteinuria (ordinal scale: 0=negative, 0.5=trace, 1=1+, 2=2+, 3=3+) shows greater proportion of HDP cases with elevated proteinuria; 41.5% of HDP cases had proteinuria ≥1+ compared to 16.0% of non-HDP cases. **(D)** Pre-pregnancy body mass index shows modest difference between groups (27.1 vs 26.1 kg/m²). Light gray bars represent women without HDP (n=106); dark gray bars represent women with HDP (n=41). All distributions overlap substantially, consistent with the moderate discriminative performance of the clinical model and reflecting the heterogeneous nature of HDP. The distributions suggest continuous relationships rather than clear thresholds for most variables.
