# Supplementary material for: Diagnostic Gap in Rural Maternal Health: Initial Validation of a Parsimonious Clinical Model for Hypertensive Disorders of Pregnancy in a Honduran Hospital
Source: Diagnostics (Basel). 2026 Jan 1;16(1):132. doi: 10.3390/diagnostics16010132 (PMC12785390; doi:10.3390/diagnostics16010132)
Supplement: Supplementary file 1 [file diagnostics-16-00132-s001.zip › figure_S2_importance.pdf]

Supplementary Figure S2. Model Feature Importance (Standardized Coefficients)

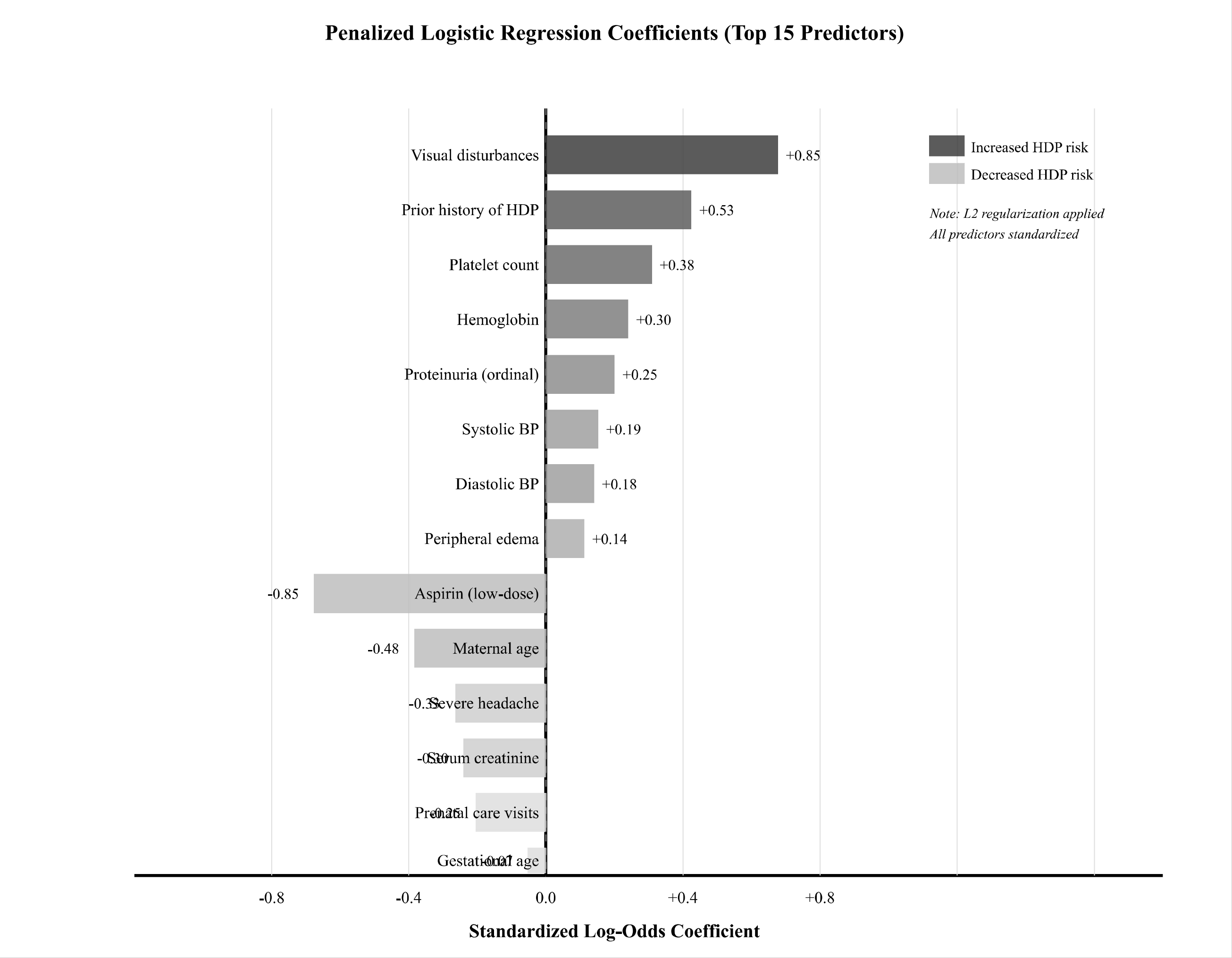

Supplementary Figure S2. Feature importance visualization showing standardized regression coefficients from the penalized logistic regression model with L2 regularization. Coefficients represent the change in log-odds of HDP per one standard deviation increase in each predictor. **Dark gray bars (positive coefficients)** indicate predictors associated with increased HDP risk, with visual disturbances showing the strongest association (+0.846). **Light gray bars (negative coefficients)** indicate protective associations, with low-dose aspirin use showing the strongest negative association (−0.846), though this may reflect both prophylactic effect and indication bias. Prior history of HDP (+0.530) shows expected recurrence risk. Notably, blood pressure variables (systolic +0.189, diastolic +0.176) show more modest coefficients than anticipated, likely due to regularization penalty and collinearity among BP measures. The counterintuitive negative coefficient for severe headache (−0.331) may reflect that other symptom combinations better discriminate HDP in this cohort. Coefficient magnitudes are affected by L2 regularization and correlation structure among predictors; absolute magnitude does not directly indicate clinical importance. All continuous predictors were standardized to zero mean and unit variance prior to modeling to enable coefficient comparison across different scales. Only the top 14 predictors by absolute coefficient magnitude are shown; complete coefficients available in Supplementary Table S5.
