# Supplementary material for: Diagnostic Gap in Rural Maternal Health: Initial Validation of a Parsimonious Clinical Model for Hypertensive Disorders of Pregnancy in a Honduran Hospital
Source: Diagnostics (Basel). 2026 Jan 1;16(1):132. doi: 10.3390/diagnostics16010132 (PMC12785390; doi:10.3390/diagnostics16010132)
Supplement: Supplementary file 1 [file diagnostics-16-00132-s001.zip › figure_S3_probabilities.pdf]

Supplementary Figure S3. Distribution of Predicted Probabilities by Outcome Status

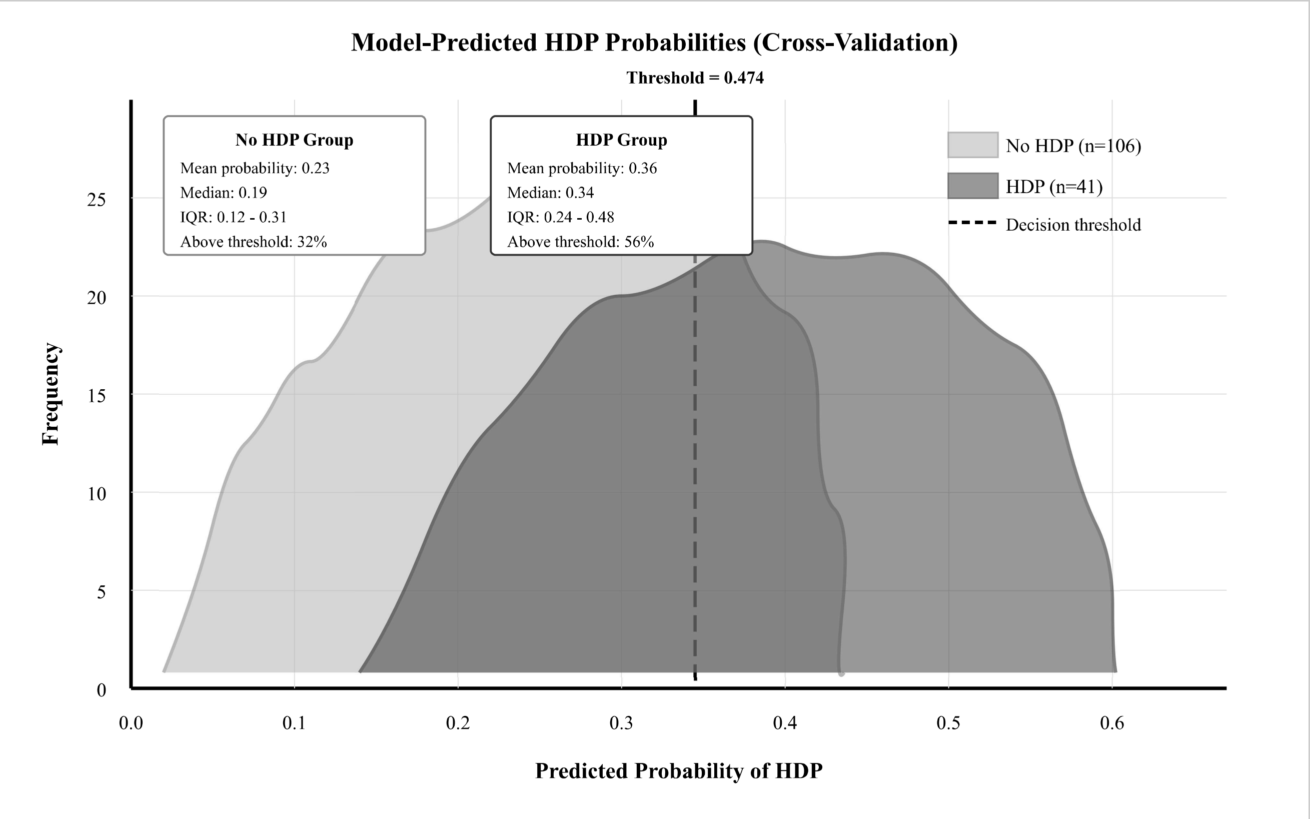

Supplementary Figure S3. Overlapping distributions of model-predicted HDP probabilities for women with (dark gray, n=41) and without (light gray, n=106) hypertensive disorders of pregnancy. The vertical dashed line indicates the decision threshold (0.474) that maximized F1-score in cross-validation. **Light gray distribution (No HDP):** Women without HDP received lower predicted probabilities on average (mean 0.23, median 0.19), with the majority of the distribution below the threshold. However, 32% of women without HDP (34 cases) received predicted probabilities above the threshold, representing false positive predictions. **Dark gray distribution (HDP):** Women with HDP received higher predicted probabilities (mean 0.36, median 0.34), with greater density above the threshold. Nevertheless, 44% of HDP cases (18 cases) received predicted probabilities below the threshold, representing false negative predictions. The substantial overlap between distributions illustrates the moderate discriminative performance (AUROC 0.614) and reflects the heterogeneous clinical presentation of HDP. Greater separation between distributions would indicate better discrimination. The distributions demonstrate that while the model provides risk stratification with modest accuracy, there is no clear probability threshold that cleanly separates cases from non-cases, necessitating clinical judgment in addition to model predictions. Probabilities are from stratified 5-fold cross-validation, ensuring independence between training and prediction.
