# Supplementary material for: Diagnostic Gap in Rural Maternal Health: Initial Validation of a Parsimonious Clinical Model for Hypertensive Disorders of Pregnancy in a Honduran Hospital
Source: Diagnostics (Basel). 2026 Jan 1;16(1):132. doi: 10.3390/diagnostics16010132 (PMC12785390; doi:10.3390/diagnostics16010132)
Supplement: Supplementary file 1 [file diagnostics-16-00132-s001.zip › figure_S4_flow_diagram.pdf]

Figure S4. Participant Flow Diagram

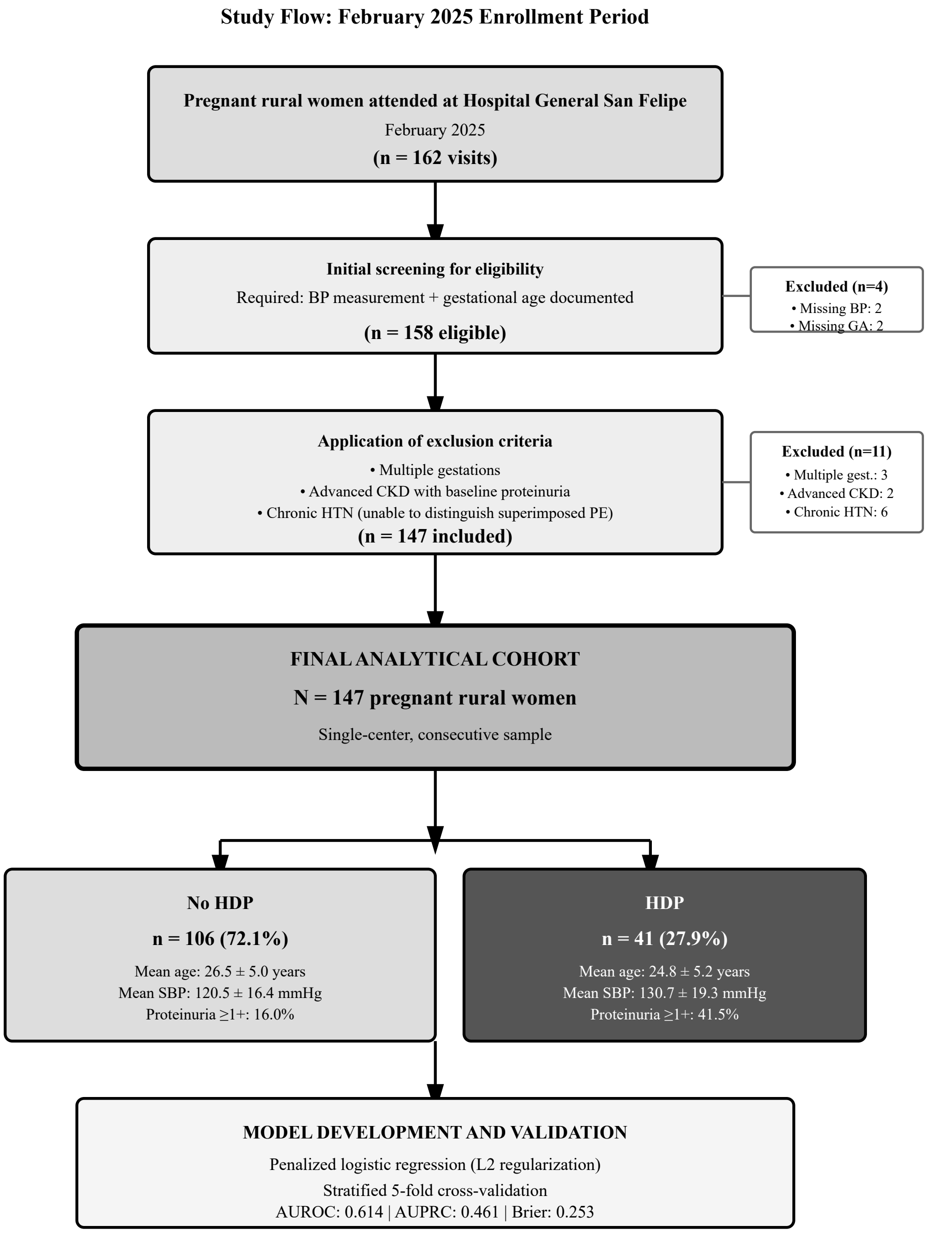

Figure S4. Participant flow diagram showing enrollment, exclusions, and final analytical cohort. All pregnant women attending Hospital General San Felipe during February 2025 (n=162 visits) were screened for eligibility. Four women were excluded due to missing essential data (blood pressure or gestational age). Eleven additional women were excluded based on predefined criteria: multiple gestations (n=3), advanced chronic kidney disease with baseline proteinuria not attributable to pregnancy (n=2), and chronic hypertension where available documentation precluded distinction between chronic hypertension alone versus superimposed preeclampsia (n=6). The final analytical cohort comprised 147 pregnant women representing the complete eligible population during the study period. Outcome adjudication identified 41 cases (27.9%) meeting criteria for hypertensive disorders of pregnancy (HDP) based on blood pressure elevation, proteinuria, and/or cardinal symptoms at the index visit. The remaining 106 women (72.1%) did not exhibit HDP. Key demographic and clinical characteristics are presented for each outcome group. All 147 women were included in model development using stratified 5-fold cross-validation, with performance metrics representing averages across folds. BP, blood pressure; CKD, chronic kidney disease; GA, gestational age; HDP, hypertensive disorders of pregnancy; HTN, hypertension; PE, preeclampsia; SBP, systolic blood pressure.
