# Supplementary material for: Diagnostic Gap in Rural Maternal Health: Initial Validation of a Parsimonious Clinical Model for Hypertensive Disorders of Pregnancy in a Honduran Hospital
Source: Diagnostics (Basel). 2026 Jan 1;16(1):132. doi: 10.3390/diagnostics16010132 (PMC12785390; doi:10.3390/diagnostics16010132)
Supplement: Supplementary file 1 [file diagnostics-16-00132-s001.zip › S1_TRIPOD+AI_Checklist.pdf]

TRIPOD+AI Checklist

Transparent Reporting of a multivariable prediction model for Individual Prognosis Or Diagnosis + AI

Manuscript Title: Diagnostic Gap in Rural Maternal Health: Initial Validation of a Parsimonious Clinical Model for Hypertensive Disorders of Pregnancy in a Honduran Hospital

Title and Abstract

Item	TRIPOD+AI Item	✓	Location in Manuscript
1	Identify the study as developing and/or validating a multivariable prediction model, the target population, and the outcome to be predicted	✓	*Title; Abstract (first sentence)*
2	Provide a summary of objectives, study design, setting, participants, sample size, predictors, outcome, statistical analysis, results, and conclusions	✓	*Abstract (structured: Background/Objectives, Methods, Results, Conclusions)*

Introduction

Item	TRIPOD+AI Item	✓	Location in Manuscript
3a	Explain the medical context and rationale for developing or validating the multivariable prediction model	✓	*Section 1, paragraphs 1-3 (maternal health disparities, HDP burden, diagnostic challenges in rural Honduras)*
3b	Specify the objectives, including whether the study describes model development, validation, or both	✓	*Section 1, final paragraph (three specific objectives stated)*

Methods: Source of Data

Item	TRIPOD+AI Item	✓	Location in Manuscript
4a	Describe the study design or source of data, and key study dates	✓	*Section 2.1 (cross-sectional study, February 2025, Hospital Gabriela Alvarado)*
4b	Specify the key study dates, including start of accrual; end of accrual; and, if applicable, end of follow-up	✓	*Section 2.1, 2.2 (February 2025 enrollment period)*
4c (AI)	Describe and justify how the AI component was developed (from scratch or existing model)	✓	*Section 2.7 (penalized logistic regression developed from scratch using scikit-learn)*

Methods: Participants

Item	TRIPOD+AI Item	✓	Location in Manuscript
5a	Specify key elements of the study setting including number and location of centres, and recruitment period	✓	*Section 2.1, 2.2 (single-center, Danlí Honduras, February 2025)*
5b	Describe eligibility criteria for participants	✓	*Section 2.2 (inclusion: BP + gestational age documented; exclusions detailed)*
5c	Give details of treatments received, if relevant	✓	*Section 2.4 (aspirin use documented as predictor)*

Methods: Outcome

Item	TRIPOD+AI Item	✓	Location in Manuscript
6a	Clearly define the outcome that is predicted by the prediction model	✓	*Section 2.3 (HDP defined: BP ≥140/90 + proteinuria or symptoms)*
6b	Report how and when outcome was assessed	✓	*Section 2.3 (index visit, retrospective chart review, clinician adjudication)*

Methods: Predictors

Item	TRIPOD+AI Item	✓	Location in Manuscript
7a	Clearly define all predictors used in developing or validating the model	✓	*Section 2.4 (demographics, obstetric history, vital signs, symptoms, labs, contextual variables - all defined)*
7b	Report when and how predictors were assessed	✓	*Section 2.4, 2.5 (index visit, chart abstraction, standardized forms)*

Methods: Sample Size

Item	TRIPOD+AI Item	✓	Location in Manuscript
8	Explain how the study size was arrived at	✓	*Section 2.2 (pragmatic consecutive sample, one-month window justified)*

Methods: Missing Data

Item	TRIPOD+AI Item	✓	Location in Manuscript
9	Describe how missing data were handled with details of any imputation method	✓	*Section 2.6 (median imputation for numerical, mode for categorical, within-fold to prevent leakage)*

Methods: Statistical Analysis Methods

Item	TRIPOD+AI Item	✓	Location in Manuscript
**10a**	Describe how predictors were handled in the analyses	✓	*Section 2.7 (standardization, one-hot encoding, ordinal as continuous)*
**10b**	Specify type of model, all model-building procedures, and method for internal validation	✓	*Section 2.7 (L2 penalized logistic regression, class weighting, 5-fold stratified CV)*
**10c (AI)**	Specify the AI method (e.g., architecture), hyperparameters, and all training and tuning details	✓	*Section 2.7 (L2 regularization, nested CV for hyperparameter tuning, inverse class weighting)*
**10d**	Specify all measures used to assess model performance and if appropriate, to compare models	✓	*Section 2.8 (AUROC, AUPRC, Brier score, calibration curves, confusion matrix at threshold)*
**10e**	Describe any model updating (e.g., recalibration) arising from validation	N/A	*Not applicable - initial development/validation only*

Methods: Risk Groups

Item	TRIPOD+AI Item	✓	Location in Manuscript
**11**	Provide details on how risk groups were created, if done	✓	*Section 2.8, 3.6 (F1-maximizing threshold identified for operational interpretation)*

Methods: Development vs. Validation

Item	TRIPOD+AI Item	✓	Location in Manuscript
**12 (AI)**	For models using supervised learning, describe how model was developed and tested	✓	*Section 2.7, 2.8 (stratified 5-fold CV, training/testing within each fold, no external validation)*

Results: Participants

Item	TRIPOD+AI Item	✓	Location in Manuscript
13a	Describe the flow of participants through the study (flow diagram recommended)	✓	*Section 3.1 (147 total, 41 HDP, 106 no HDP; exclusions described in Methods 2.2)*
13b	Describe characteristics of participants, and number with missing data for predictors and outcome	✓	*Section 3.1, 3.2, 3.3; Table 1 (demographics, clinical variables, lab availability noted)*
13c (AI)	For each dataset, provide details on how it was obtained and preprocessed	✓	*Section 2.5 (chart abstraction), 2.6 (missing data), 2.7 (preprocessing: standardization, encoding)*

Results: Model Development

Item	TRIPOD+AI Item	✓	Location in Manuscript
14a	Specify number of participants and outcome events in each analysis	✓	*Section 3.1, Table 1 (N=147, 41 events, stratified by outcome)*
14b	Report unadjusted associations between predictors and outcome	✓	*Section 3.1, 3.2, 3.3; Table 1 (means by group showing differences in BP, proteinuria, etc.)*

Results: Model Specification

Item	TRIPOD+AI Item	✓	Location in Manuscript
15a	Present the full prediction model to allow predictions for individuals	✓	*Section 3.7; Table 3 (coefficients for all predictors); equation implicit in logistic regression*
15b	Explain how to use the prediction model	✓	*Section 4.3 (operational interpretation, threshold usage for triage)*
15c (AI)	For AI models, describe the trained model to allow full reproducibility or provide access	✓	*Section 2.7 (scikit-learn implementation specified); Data Availability (code available on request)*

Results: Model Performance

Item	TRIPOD+AI Item	✓	Location in Manuscript
16	Report performance measures with confidence intervals	✓	*Section 3.4, 3.6; Table 2 (AUROC, AUPRC, Brier, sensitivity, specificity, PPV, NPV with SD across folds)*
17 (AI)	For AI models, describe measures to handle overfitting and results of such measures	✓	*Section 2.7 (L2 regularization), 2.8 (stratified k-fold CV); Section 3.4 (CV performance reported)*

Results: Model Updating

Item	TRIPOD+AI Item	✓	Location in Manuscript
18	If model updating was done, report updated model specification and performance	N/A	*Not applicable - initial development study only*

Discussion

Item	TRIPOD+AI Item	✓	Location in Manuscript
19a	Discuss implications of model performance	✓	*Section 4.1, 4.3 (moderate performance, clinical utility for rule-out, triage applications)*
19b	Give overall interpretation considering objectives, limitations, results, and other studies	✓	*Section 4.1, 4.2, 4.4 (comparison with literature, contextual interpretation for rural settings)*
20	Discuss limitations, including potential bias or imprecision	✓	*Section 4.4 (seven limitation categories: cross-sectional, sample size, outcome misclassification, missing data, single-center, no clinical impact assessment)*
21 (AI)	Discuss potential biases and fairness issues for AI models	✓	*Section 1 (algorithmic bias, health equity); Section 4.3 (health equity implications); Section 4.4 (selection bias from missing data)*

Other Information

Item	TRIPOD+AI Item	✓	Location in Manuscript
22a	Provide information about availability of supplementary resources	✓	*Data Availability Statement (code and de-identified data available on request)*
22b (AI)	Provide information on how trained model can be accessed and used	✓	*Data Availability Statement (analysis code available; model parameters in Table 3)*
23	Give source of funding and role of funders	✓	*Funding section (no external funding)*
24	Declare authors' conflicts of interest	✓	*Conflicts of Interest section (no conflicts declared)*

Checklist Completion: 100% (all applicable items addressed)

This checklist follows the TRIPOD+AI guidelines published in BMJ 2024;385:e078378
Collins GS, Dhiman P, et al. TRIPOD+AI statement: updated guidance for reporting clinical prediction models
